# Scandal of Out-Patients

**Published:** 1911-10-14

**Authors:** John Hy. Johnson

**Affiliations:** Royal Westminster Ophthalmic Hospital, King William Street, West Strand, W.C.


*To the Editor of* The Hospital.

### SCANDAL OF OUT-PATIENTS.

*To the Editor of* The Hospital.

Sir,—I have read with much pleasure your Special number, and especially the article "The Scandal of Out-Patients." With regard to the paragraph in the above, which states that many of the out-patient departments are ill-lighted, badly ventilated, etc., I should like your representative to call here, and inspect the new out-patient waiting hall and the improvements generally which have been carried out at this institution during the past twelve months for the benefit of our numerous patients. Full particulars of the scheme of improvement are given in our annual report, of which a copy is enclosed. I am, yours faithfully,

John Hy. Johnson.

Royal Westminster Ophthalmic Hospital,
King William Street, West Strand, W.C.
October 11, 1911.

